# Towards a standardized diabetic prolonged wound healing model in hairless SKH1 mice

**DOI:** 10.3389/ebm.2026.10857

**Published:** 2026-03-20

**Authors:** Elle Koivunotko, Julia Monola, Chris S. Pridgeon, Jere Linden, Riina Harjumäki, Emrah Yatkin, Mari Madetoja, Marjo Yliperttula

**Affiliations:** 1 Division of Pharmaceutical Biosciences, Drug Research Program, Faculty of Pharmacy, University of Helsinki, Helsinki, Finland; 2 Finnish Centre for Laboratory Animal Pathology, HiLIFE, University of Helsinki, Helsinki, Finland; 3 Department of Veterinary Biosciences, Faculty of Veterinary Medicine, University of Helsinki, Helsinki, Finland; 4 Central Animal Laboratory, University of Turku, Turku, Finland; 5 Made Consulting Ltd, Turku, Finland

**Keywords:** collagen deposition, diabetogenic SKH1 mice, *in vivo* prolonged wound healing model, macrophage activity, STZ induction

## Abstract

Chronic wounds, particularly those associated with diabetes, pose a significant clinical challenge due to their impaired healing dynamics and lack of reliable and standardized preclinical models. This pilot study aimed to establish a diabetogenic, immunocompetent, hairless mouse model (SKH1 strain) to simulate prolonged wound healing. Diabetes was induced by streptozotocin administration, followed by the creation of full-thickness dorsal skin wounds. Wounds were treated with either saline or nanofibrillated cellulose hydrogel as a model treatment. Wound healing progression and blood glucose were monitored, and histopathological assessments were performed after a 14-day experiment. In addition, for the first time, the Thermidas thermal imaging system was used in an *in vivo* mouse model to evaluate skin temperature. Results demonstrated that diabetes induction successfully prolonged wound closure by 5 days compared with the previously described acute wound model in the same strain with the identical protocol without streptozotocin (STZ) induction. Histopathological analyses showed increased macrophage activity (16.2% vs. 2.2% in the treatment groups and 10.2% vs. 0.3% in the control groups) and decreased collagen deposition (12.2% vs. 43.2% in the treatment groups and 17.6% vs. 27.4% in the control groups), suggesting prolonged wound healing. These findings support the use of hairless SKH1 mice as a viable model for studying prolonged diabetic wound healing and evaluating future therapeutic candidates.

## Graphical Abstract

**Figure:**
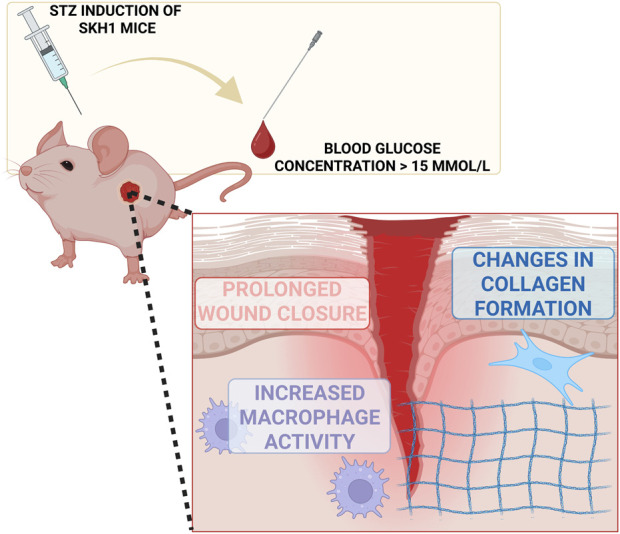

## Impact statement

Chronic wounds are often referred to as a silent epidemic and represent a growing global challenge with increasing prevalence each year. The development of novel bioactive wound therapies is hindered by the lack of standardized pre-clinical models and limited understanding of prolonged wound-healing pathophysiology. In this study, together with our previous work, we provide new insights into the potential of the SKH1 nude mouse strain as a versatile model for both acute and chronic wound healing research. To our knowledge, this is the first methodological study directly comparing diabetogenic and healthy SKH1 mice in a full-thickness wound model, revealing important differences in wound-healing pathology and treatment candidate efficacy. Publication of this work offers the opportunity to advance more standardized, reproducible wound-healing models that can accelerate therapeutic development while adhering to the principles of the 3Rs.

## Introduction

An aging population and the rising prevalence of chronic diseases, such as diabetes and cardiovascular disorders, present global challenges in managing chronic wounds. Chronic wounds have been reported to cost up to 97 billion USD annually [1] and increase the risk of mortality, particularly in patients with diabetic foot ulcers [2]. Insufficient homecare and the development of antibiotic resistance have led to a vicious cycle of chronic wound treatment [3], which will not be resolved without novel treatments and a better understanding of wound pathophysiology.

Diabetes is a leading systemic contributor to chronic wound development, primarily by inducing local ischemia, elevating protease activity, and impairing skin cell function due to persistent hyperglycemia [4, 5]. While the current standard of care (including wound cleansing, pressure offloading, and infection control) remains essential, alternative therapies are being explored to enhance oxygen delivery, stimulate angiogenesis, and restore cellular activity. These include negative pressure wound therapy, bioactive hydrogels, and the application of growth factors [6, 7]. However, despite their promise, these approaches have yielded inconsistent results and lack standardized protocols, limiting their integration into routine chronic wound management.

The development of effective wound therapies is hindered by the heterogeneity of chronic wounds and the complexity of the wound healing process, making it difficult to establish standardized and reproducible preclinical models. Acute and chronic wounds differ significantly in key biological processes, further complicating both *in vitro* and *in vivo* modeling. The classification of wounds as acute or chronic is primarily determined by their healing duration, which itself depends heavily on the wound’s original size and location [8]. A wound is considered chronic if it fails to progress through the four partially overlapping healing phases (hemostasis, inflammation, proliferation, and maturation) within approximately 12 weeks. Beyond delayed or incomplete closure, chronic wounds are also characterized by persistent inflammation and impaired cellular function, often influenced by local or systemic conditions such as chronic diseases or infections [9]. In diabetes, for instance, angiogenesis—the formation of new blood vessels from existing vasculature—is impaired, resulting in insufficient oxygen and nutrient delivery to the wound site and contributing to delayed healing [10]. This vascular deficiency disrupts the balance of growth factors and cytokines, impairs epithelialization, and delays wound closure [11, 12]. Elevated levels of reactive oxygen species and altered macrophage activity lead to prolonged inflammation, as macrophages in chronic wounds fail to undergo the normal transition from pro-inflammatory (M1) to pro-regenerative states (M2) [13]. This sustained inflammation negatively impacts the formation of fibrous tissue and angiogenic activity [14]. Additionally, later stages of healing, including proliferation and maturation, are disrupted. Increased metalloproteinase activity and altered fibroblast function lead to an imbalance in collagen production [15, 16]. Such disturbances can result in excessive or pathological scarring, for which no definitive treatment currently exists.

Although research has focused on reducing the use of *in vivo* models with versatile three-dimensional *in vitro* models, the use of animals remains the gold standard in medical device and medicinal drug development for wound treatment. From the anatomical perspective, porcine models provide the most similar skin structure to human skin when comparing skin thickness, layers, and hair density [17]. Conversely, a more standardized genetic background and established genetic modifications, lower cost, and simpler habitat maintenance mean that mouse models are the most common *in vivo* model. However, this causes difficulties when translating *in vivo* findings to the clinic due to the larger differences between mice and humans, such as differences in skin thickness and contraction and its attachment to underlying tissue [18]. The most common method to study diabetic wounds *in vivo* is with streptozotocin (STZ)-induced mice [19]. STZ is a chemotherapeutic agent for the treatment of pancreatic β -cell carcinoma, the cells in which normal insulin production occurs. In mice, STZ causes necrosis of the β -cells, decreasing insulin production as in type I diabetes [20]. Although STZ induction is widely used, the protocols differ substantially between laboratories in terms of the experimental setups, e.g., variations in STZ dosing protocol, animal strain, experimental endpoints, animal diet, and data monitoring. Additional challenges come from the lack of both acute and prolonged wound healing models in the same experimental modality, challenging the understanding of the pathological differences.

Although most studies using STZ induction in rodents are considered disease pathology models, the model has also been used in chronic wound healing research. However, most models are generated with strains with typical skin and hair, which decreases the similarity to humans [21]. This challenge can be addressed by using hairless mouse strains, where the lack of hair and hair-follicle–derived wound-healing stem cells may offer a more suitable foundation for developing an *in vivo* wound healing model [22, 23]. Although these strains retain certain biological limitations, like differences in skin structure [24] and the wound closure process [25, 26], they allow for easier wound creation, more consistent monitoring of the healing process, and improved capacity for thermographic assessment compared with other strains. Currently, there are few chronic wound models with immunocompetent hairless mouse strains, which encouraged us to generate a prolonged wound healing model in hairless SKH1 mice using a standardized STZ induction protocol. In addition, our previous study using SKH1 mice to generate an acute full-thickness wound model with an identical protocol to evaluate the efficacy and safety of wound treatment candidates was successful; it was carried out with good animal welfare, simplified wound creation, good monitoring of wound healing, and evaluation of possible changes in the skin during the experiment.

In this pilot study, our aim was to produce a tractable prolonged full-thickness wound healing model using STZ-induced hairless SKH1 mice, which has been used previously in our acute wound model study without diabetes induction [27]. For brevity, data in the prolonged wound healing model are henceforth referred to as “chronic wounds”. Based on this study we emphasize the following: 1) the potential for future standardization of current *in vivo* wound models by using the same strain and experimental set up in both acute and prolonged wound healing experiments; 2) the use of non-invasive monitoring tools to evaluate the progression of wound healing and possible infection with thermograph analysis; and 3) the use of hairless mice for simplified wound monitoring, and avoidance of ethically contentious methods such as mechanical prevention of wound closure. Through comparison with data from the previous acute wound model, changes in wound healing physiology, including epithelialization, inflammation, angiogenesis, and collagen deposition, could be observed in response to the same treatments. We hypothesize that hyperglycemic SKH1 mice with prolonged wound healing characteristics, including longer wound closure time, induced macrophage activity, and reduced collagen deposition, can be produced using STZ induction. By considering both acute and prolonged wounds, this study indicates the importance of standardized preclinical models for future wound care development.

## Materials and methods

### Model treatment formulations

To illustrate the effects of the material candidate in the chronic wound model, medical-grade nanofibrillated cellulose (NFC) hydrogel (FibGel, UPM Biomedicals) [2.9% (m/v)] was used as a model treatment as in our previous publication [27]. NFC hydrogel was diluted into 0.8% (m/v) with sterile water before use. 0.9% (m/v) NaCl was used as a control treatment.

### Streptozotocin injection

Streptozotocin (STZ) (Sigma-Aldrich, batch 0661505.4, MO, USA) was solubilized in NaCl 0.9% (batch 214328131, Braun) into a final concentration of 8.0 mg/mL. STZ was prepared fresh daily and injected intraperitonially within 15 min of preparation. STZ bolus (40 mg/kg body weight) was administered daily for 5 consecutive days as previously described [28] using a Myjector 27Gx1/2″ Terumo 0.3–0.5 mL insulin syringe. The dosing volume was 5 mL/kg. The injection site of skin was wiped with 70% EtOH prior to injection. Animals were not fasted before STZ injection to avoid additional stress factors for mice.

### Animals

STZ induction was performed on 10 male SKH1 mice (Crl:SKH1-Hrhr, SPF, Charles River) at age 8–9 weeks. In total, nine of the STZ-induced mice underwent the chronic wound model. The pilot animal experiments were approved by the National Project Authorization Board of Finland (license number ESAVI-25539-2024), and the study conformed to the following guidelines: DIRECTIVE 2010/63/EU of the European Parliament and the Council, Finnish Act (497/2013), Government Decree on the Protection of Animals Used for Scientific or Educational Purposes (564/2013), and Guidance document on the Recognition, Assessment and Use of Clinical Signs as Humane endpoints for Experimental Animals Used in Safety Evaluation, Environmental Health and Safety Monograph Series on Testing and Assessment (No 19. OECD 2000). The reporting of this animal research followed the ARRIVE 2.0 (Animal Research: Reporting *In Vivo* Experiments) guidelines [29] in the spirit of the pilot study.

This non-Good Laboratory Practices (GLP) animal experiment was performed in a GLP-certified Central Animal Laboratory, Turku University, Finland. The acclimatization period before the first experiment procedure was 6–13 days. The first STZ injection was given after the acclimatization period, and injections were given on 5 consecutive days. The mice were housed four to five animals per cage before experimentation and individually during the experiments, starting on study day 0. Cellulose paper and cardboard houses were used as environmental enrichment. Laboratory room temperature was 21 °C ± 3 °C, relative humidity was between 40% and 60%, and artificial lighting followed a 12-hour light, 12-hour dark cycle. A laboratory rodent chow diet (Teklad2920, Inotiv) and water were offered *ad libitum,* and the animals were cared for according to the standard operating procedures of Central Animal Laboratory. The clinical status was checked twice daily during the experiment period. The animals were weighed on study days 0, 2, 5, 6, 8, 10, 12, and 14 after wound surgery without dressing.

### Blood glucose monitoring

The blood glucose of the mice was measured for the first time after the first STZ injection using the ACCU Check Aviva glucose meter and Accu-Chek Aviva Blood Glucose Test Strips (lot 690720). The validity of the meter was checked by using Accu-Chek Guide control solutions (lot 24700675). Blood for glucose determination was taken from the tail vein by needle prick using a 27G needle and analyzed with a glucometer (0.6 µL of blood). When the blood glucose level was ≥15 mmol/L (corresponding to 270 mg/dL) in two consecutive measurements, a mouse was diagnosed with diabetes.

When blood glucose rose to ≥20 mmol/L, blood glucose was monitored daily, and insulin treatment was started at ≥25 mmol/L. The insulin doses were administered to mice once or twice a day, depending on the glucose level response (Lantus 100 IU/mL, 0.5 IU accuracy, batch 3F231A, insulin pen JuniorSTAR). The starting dose was 0.5 IU per mouse per day, after which the dose varied between 0.5 IU and 3.0 IU (Table 1).

**TABLE 1:** Glucose levels and the used insulin treatment dose.

Glucose level	Insulin treatment/day
<20 mmol/L	No insulin
20–25 mmol/L	0.5 IU
25–30 mmol/L	0.5–2 IU
>30 mmol/L	1–3 IU

### Surgical procedure

Full-thickness wounds were created as described previously [27]. Briefly, 22 days after the last STZ injection, mice were preoperatively given a subcutaneous injection of buprenorphine (Bupaq multidose vet 0.1 mg/kg, Ritchter Pharma) and carprofen (Rimadyl vet 16 mg/kg, Zoetis) and anesthetized before the surgical procedure with isoflurane (3.5% Attane Vet 1,000 mg/g). Prior to incision, infiltrative local anesthesia of lidocaine (Lidocaine 4 mg/kg, Baxter) was applied under the skin. Rimadyl was also administered 6–8 h after surgery and every 10–13 h on study days 1 and 2 as a postoperative treatment. Surgical sites were disinfected with a skin disinfectant, and incisions were made on both sides of the animal’s back with sterile scissors and tweezers, first to the right side and then to the left side. The veterinarian was blinded from the dosing sites of the treatment. The wounds were measured, and photographs were taken immediately after surgery. After applying the model treatments on the right side and saline as a control on the left side, wounds were covered with transparent, non-occlusive polyurethane film.

A total of nine mice underwent surgery since one animal was hypoglycemic on the operation day (blood glucose <1.0 mmol/L) and had a decreased level of consciousness. The animal was treated with 0.5 mL of 10% sugar-water solution (D-sucrose, p.o.) and honey on the mouth mucous membranes. After 1 h, the animal was given 0.2 mL of 50% sugar solution. After 50 min of the last sugar application, the blood glucose level was 4.7 mmol/L. Subsequently, 10% sugar solution was provided *ad libitum*. After 3 h, the blood glucose level was 24.0 mmol/L. Blood sugar levels and insulin treatment were later performed in the same way as with animals that underwent surgery for welfare purposes, but are not plotted in the figures.

### Wound monitoring

The length and width of the wounds were measured immediately after their creation and on study days 2, 5, 6, 8, 10, 12, and 14 using our previously published protocol with a calibrated digital caliper (Mitutoyo 0–150 mm) [27]. The analyst measured the wounds as a one-time method to keep the objectivity of the wound measurements. The wound area was calculated with the following equation according to Moreira et al. [30]:

$$\left(\text{Wound length}/2\right)\;\times \;\left(\text{wound width}/2\right)\;\times \;\pi$$ (1)

At the end of the study, mice were weighed, all macroscopic abnormalities at the wound site were recorded, and photographs of the wounds were taken. Blood samples (ca. 600 μL) were taken by heart puncture under isoflurane anesthesia in K2E Microtainer tubes (Becton Dickinson, United States, NJ, lot 4174515) for hematological analyses, which were implemented as blinded with sample coding without identifying the wound. Hematology test analyses were performed in EDTA blood tubes according to the protocol of Central Animal Laboratory using VetScan HM5 hematological analyses (Abaxis, United States). A piece of skin (1 cm^2^), including the wound site, was removed and placed into 10% phosphate buffered neutral formalin (Oy Reagena Ltd, Finland, lot CB19/1). Subsequently, tissue samples were embedded in paraffin and cut into 4 µm sections for further histopathological analyses.

### Thermal monitoring of the wounds

For wound temperature monitoring, a Thermidas IRT-384 Tablet (Vet VistaClinic, Software 1.4.2, Thermidas Oy, Tampere, Finland) was used. Images were taken in the same operating room (ambient temperature of 21–23 °C) within a 2-hour period on days 0, 2, 5, 6, 8, 10, 12, and 14 at the same time as the weight and wound measurements. Before imaging the mice, a blank image from the operating room table was taken to calibrate the temperature range. Images were taken approximately 15 cm from the animal. Animals were anesthetized before imaging, and the total operation time was approximately 4 min per animal. The thermal resolution of the images was 384 × 288 pixels. The temperature of the wounds was compared to healthy skin on the same animal.

### Histopathology and immunohistochemistry

Wound tissue sections (4 µm thickness) were stained with hematoxylin and eosin (HE) for histological examination. A veterinary pathologist (J.L.) blinded to the sample identity assessed the staining. Masson’s trichrome (MT) staining was used to evaluate collagen deposition by calculating the blue color intensity of the wound area.

Immunostaining was performed as reported previously by Koivuniemi et al. [31] and Koivunotko et al. [27]. Briefly, tissue sections were deparaffinized (3 × 2 min in xylene, 2 × 10 min in 100% ethanol, 2 × 10 min in 94% ethanol and 2 × 5 min in distilled H_2_O). Antigen retrieval was carried out in 10 mM citrate buffer (Merck, Germany) with 0.05% Tween 20 solution (Merck) (pH 6) at 99 °C for 3 × 10 min. After antigen retrieval, endogenous peroxidase activity was blocked with 3% H_2_O_2_ (Merck) for 10 min. Sections were then blocked for 1 h in 5% bovine serum albumin (BSA, Merck) in Tris-buffered saline with Tween 20 (TBS-T, Merck). Next, sections were blocked with endogenous biotin (Avidin/Biotin blocking kit, Vector Laboratories, CA, USA) for 15 min with Avidin first and then 15 min with Biotin.

Tissue sections were incubated overnight at 4 °C with anti-rabbit CD31/platelet endothelial cell adhesion molecule (CD31/PECAM-1, 1:100, CAT: NB100–2284, Novus Biologicals, UK) or 1 h at room temperature with anti-rabbit ionized calcium-binding adaptor molecule 1 (Iba-1, 1:500, CAT: 019-19741, FujiFilm, USA) or anti-rabbit lysozyme (LZM, 1:1000, CAT: A0099, Agilent, CA, USA) as a primary antibody in 3% BSA/TBS-T. After washing with TBS-T, tissue sections were stained for 1 h at room temperature with goat anti-rabbit IgG as biotinylated secondary antibody (1:1000 for CD31 and 1:200 for Iba1 and LZM, Abcam, UK) in 3% BSA/TBS-T. VECTASTAIN Elite ABC reagents (Vector Laboratories) were used for antibody detection by staining the tissue sections for 30 min at room temperature, after which they were treated with 3,3′-diaminobenzidine (DAB) HRP substrate treatment (Vector Laboratories). All stained tissue sections were counterstained with hematoxylin, dehydrated, and covered with cover clips using a mounting medium (Coverquick 2000, VWR International, PA, USA).

Histoscanning was performed with a Pannoramic 250 Flash III brightfield digital slide scanner (3DHISTECH Ltd., Hungary) at the Histoscanner core facility (University of Helsinki) using the updated coding from the original one to increase the blindness of the image analyses. The thickness and length of the neo-epithelium and dermis were measured with CaseViewer (3DHISTECH Ltd., version 2.4). The intensity of the stained fibrous connective tissue and macrophage infiltration was evaluated using object and pixel classifications in QuPath 0.4.3 software [32]. To compare the results from the prolonged wound healing model with the previously described acute wound model using the same histopathological staining protocol [27], acute wound tissue section analyses were repeated with the same threshold settings. Thresholds in different analyses were as follows: 0.4 for iba-1 and LZM staining, 0.15 for CD-31 staining, 0.64 for MT staining.

### Statistical analyses

The data are presented as mean standard deviation (STDEV). For the normally distributed data, statistical significance was determined with an unpaired t-test for two variables and a one-way ANOVA and Tukey HSD *post hoc* test for others. To evaluate the effects of glucose levels [defined as low (<23.4 mmol/L) and high (>23.4 mmol/L)] on the results, multivariate analyses were performed with a Wilks’ Lambda test. Significance was concluded when *p < 0.05, **p < 0.01, ***p < 0.001, or ****p < 0.0001.

## Results

### Diabetes was successfully induced in all 10 animals

**FIGURE 1:**
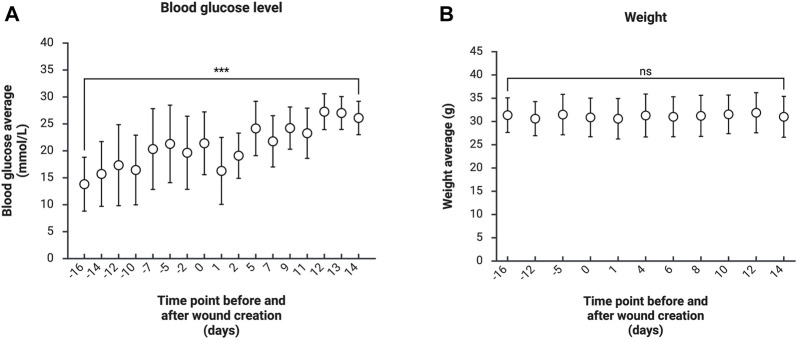
Mean blood glucose concentration (n = 9) **(A)** and mean weight (n = 9) **(B)** of animals throughout the experiment before and after surgery. Error bars indicate STDEV, and significance was calculated using one-way ANOVA (ns: p ≥ 0.05, ***p < 0.001).

Diabetes was induced in all animals (blood glucose level $\left.\geq 15\text{ mmol}/L\right)$ within 21 days after the first STZ bolus injection. In this study, animals were not fasted before STZ injections or before blood glucose measurements. The blood glucose measurements were performed daily or twice daily to ensure animal welfare and titrate the insulin dose for each animal. The mean blood glucose level on the last experimental day was 27 mmol/L (STDEV 4.1 mmol/L), which was significantly increased (p < 0.001) compared to the 16 days prior to surgery (Figure 1A). Based on clinical monitoring and weight (Figure 1B), even animals with high blood glucose levels (>30 mmol/L) were in good condition during the whole experiment. The non-significant weight decrease (3–9%) in some animals was correlated with higher blood glucose (>23.4 mmol/L) but was insignificant in multivariate analyses (p = 0.085).

Hematological analyses showed increased values in most of the measured parameters (Table 2) when compared with the acute wound model [27]. In the case of hematocrit, mean corpuscular hemoglobin, plateletcrit, and the total concentration of monocytes and neutrophils, no changes (less than 0.5-unit difference) were observed between the models. On the other hand, mean corpuscular volume, red blood cell distribution, and percentage of lymphocytes were decreased. However, no clinically relevant differences were observed.

**TABLE 2:** Hematological analyses of total blood samples after a prolonged wound healing model experiment. The results from the previous acute wound model in SKH1 [27] with informed reference values are presented on the right-hand side of the table.

Parameter	Unit	Value, mean (STDEV n = 9)	Previous acute wound model value (n = 3–6)	Reference value [33–35]
WBC- White blood cell	10^9^/L	5.6 (2.5)	4.1 (1.5)	9.3 (1.8)
RBC- Red Blood Cell	10^12^/L	11.1 (0.3)	9.9 (2.1)	9.4 (0.4)
HGB- Hemoglobin	g/L	157.6 (7.5)	142 (186.7)	120 (10.2)
HCT- Hematocrit	%	52.4 (4.0)	52.9 (4.1)	49.4 (2.2)
MCV- Mean Corpuscular Volume	fl	47.2 (3.0)	53.7 (2.3)	53.9 (2.4)
MCH- Mean Corpuscular Hemoglobin	pg	14.2 (0.4)	14.4 (0.6)	17.1 (0.4)
MCHC- Mean Corpuscular Hemoglobin Concentration	g/L	301.9 (15.5)	268 (16.6)	317.4 (9.8)
PLT- Platelet	10^9^/L	431.2 (95.8)	335 (36.5)	285–890
PCT- Plateletcrit	%	0.3 (0.1)	0.2 (0.02)	N/Av
MPV- Mean Platelet Volume	fl	6.6 (0.4)	6.1 (0.9)	4.6 (0.1)
PDWs- Platelet Distribution Width	fl	7.4 (0.4)	6.4 (1.8)	7.7 (1.1)
PDWc- Platelet Distribution Width	%	28.4 (0.7)	26.6 (3.2)	N/Av
RDWs- Red blood cell Distribution	fl	32.7 (1.7)	38.3 (1.2)	29.1 (3.3)
RDWc- Red blood cell Distribution	%	21.6 (0.5)	19.9 (0.8)	16.4 (0.4)
LYM- Lymphocytes	10^9^/L	4.1 (2.1)	3.6 (1.7)	5.7 (1.3)
MON- Monocytes	10^9^/L	0.2 (0.1)	0.2 (0.1)	0.6 (0.2)
NE- Neutrophils	10^9^/L	1.3 (0.6)	1.0 (0.1)	N/Av
LY%- Lymphocytes	%	71.9 (7.3)	73.8 (3.4)	61.5 (6.9)
MO%- Monocytes	%	4.8 (2.2)	4.9 (2.5)	6.1 (1.3)
NE%- Neutrophils	%	23.3 (6.0)	21.3 (10.2)	N/Av
EOS- Eosinophils EO%- Eosinophils BAS- Basophils BA%- Basophils	10^9^/L % 10^9^/L %	0.0	0.0	N/Av 0.6 (0.2) 0.0 (0.0) 0.0 (0.0)

### Wounds were closed by day 14

**FIGURE 2:**
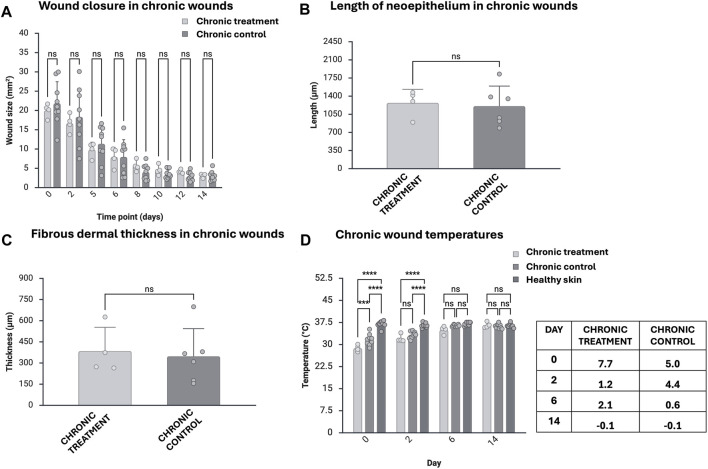
Measurements of wound size over time (n = 4 in treatment group, n = 9 in control group) **(A)**, mean length of neo-epithelium (n = 4 in treatment group, n = 6 in control group) **(B)**, mean thickness of dermal fibrous tissue (n = 4 in treatment group, n = 6 in control group) **(C)**, and mean wound site temperature (n = 4 in treatment group, n = 9 in control group) with temperature differences (°C) compared with healthy skin presented in a table **(D)**. Error bars indicate STDEV, and significance was calculated using unpaired t-test and one-way ANOVA (ns: p ≥ 0.05, ***p < 0.001, ****p < 0.0001).

The endpoint of the experiment was on day 14, during which the first closed wounds were observed (<5 mm^2^) (Equation 1). No significant differences in wound closure, epithelialization, or fibrous tissue thickness between model treatment and control groups were observed (Figures 2A–C). The mean epithelium length in tissue samples was 1271 µm in treated wounds and 1207 µm in controls. The mean fibrous tissue thickness measured from the dermis site was 385 µm in treated wounds and 348 µm in control wounds. Histological evaluation of the HE-stained sections by the pathologist (J.L) showed regrown epidermis to cover the wound areas in all mice, often exhibiting minimal to mild epidermal hyperplasia. Model treatment was present extracellularly as large homogenous translucent depots occupying over half of the wound area in most treated samples.

The wound area treated with model treatment showed significantly decreased temperature during the first week compared with healthy skin temperatures (8 °C lower temperature on day 0, 2 °C on day 6, Figure 2D; Supplementary Material 1). In addition, treated wounds were 3 °C colder compared with control wounds on day 0.

### Blood vessel formation at the wound site

**FIGURE 3:**
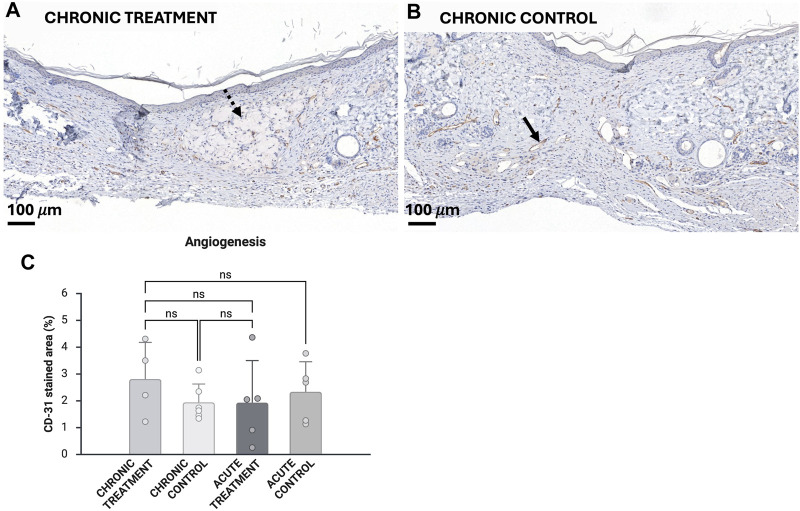
The contrast measurements of CD31-stained vessels in treated chronic wounds **(A)** and chronic control wounds **(B)**. The dotted arrow indicates model treatment (nanofibrillated cellulose hydrogel), and the solid arrow indicates an example of a stained vessel. Percentual area of the CD31-stained vessels (n = 4 for chronic treatment, n = 6 for chronic control) **(C)**. Analyses were also performed for previously obtained histopathological samples (n = 5 for acute treatment, n = 5 for acute control) [27]. Scale bar 100 μm. Error bars indicate STDEV, and significance was calculated using one-way ANOVA (ns: p ≥ 0.05).

The presence of newly formed blood vessels was measured from CD31 stained histopathological samples. The percentual contrast area of the CD31 staining in wounds was >5% in all wounds (Figure 3). In addition, the analyses were also performed for previous histopathological samples from the acute wound model on day 9 [27], but no significant differences were observed between CD31 stained tissue samples. In wounds with model treatment, standard deviation was higher than in control groups.

### Increased infiltration of macrophages with higher activity in prolonged wound healing model

**FIGURE 4:**
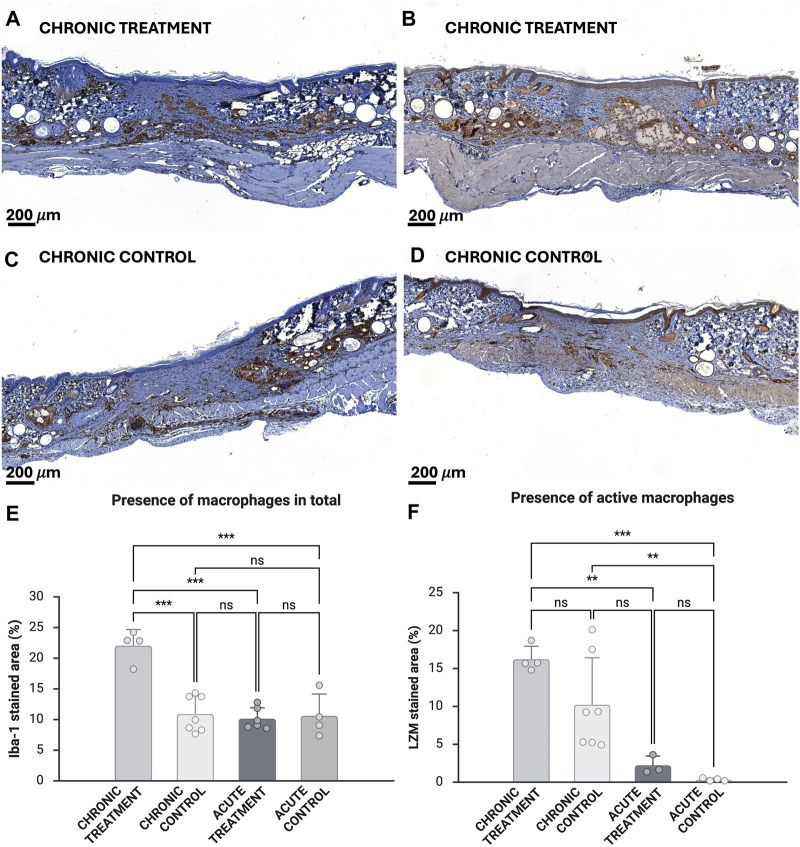
The presence of macrophages in total stained with Iba-1 **(A,C)** and active macrophages stained with LZM **(B,D)**. Upper tissue sample images are from treated chronic wounds and lower ones from chronic control wounds. Percentual area of the iba-1 stained macrophages (n = 4 for chronic treatment, n = 6 for chronic control, n = 6 for acute treatment, n = 4 for acute control) **(E)** and LZM stained macrophages (n = 4 for chronic treatment, n = 7 for chronic control) **(F)**. Analyses were also performed for previously obtained histopathological samples (n = 3 for acute treatment, n = 4 for acute control) [27] **(C)**. Scale bar 200 µm. Error bars indicate STDEV, and significance was calculated using one-way ANOVA (ns: p ≥ 0.05, **p < 0.01, ***p < 0.001).

Macrophage cell infiltration was evaluated with both total macrophage staining (Iba-1) and active macrophage staining (LZM) (Figures 4A–D). The contrast analyses were also performed for previously obtained Iba-1 and LZM stained histopathological samples from the acute wound model on experimental day 9 [27] (Figures 4E,F). The treated chronic wound showed the highest presence of macrophages at the wound site [24.8% ( ± 6.6%)]. Despite the treatment conditions, chronic wounds showed significantly increased presence of macrophages with higher activity [10.2 ( ± 6.2%) and 16.23% ( ± 1.7%)] when compared with acute wounds [0.3% ( ± 0.2%) and 2.2% ( ± 0.9%); p = 0.003 between treatment groups, p = 0.009 between control groups, p = 0.0005 chronic treatment vs. acute control].

In the histological examination the treated wounds showed a stereotyped moderate inflammatory reaction, consisting of a thin rim of macrophages surrounding and infiltrating the hydrogel depots and single macrophages that contained modest amount of foamy hydrogel material as well as a moderate macrophage and lymphocyte infiltrate outside of the depots. Very few multinucleated giant cells were present. In comparison, most control wounds displayed mild to moderate mononuclear inflammatory cell infiltrate in the wound area.

Notably, most samples, regardless of treatment, exhibited pronounced, focally extensive hypodermal and deep dermal pyogranulomatous inflammation affecting damaged hair follicles and sebaceous glands (Supplementary Material 2). Granuloma formation was most prevalent at the wound borders and displayed a striking increase compared to the acute wound model [27].

### Collagen deposition was slowed down in prolonged wound healing model

**FIGURE 5:**
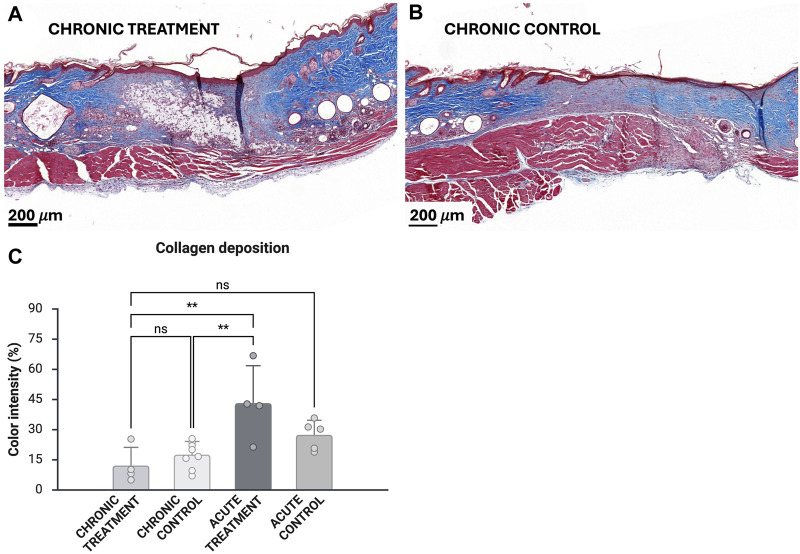
Masson trichrome analyses of treated chronic wounds **(A)** and control wounds **(B)**. Collagen deposition was measured from blue staining intensity (n = 4 for chronic treatment, n = 7 for chronic control) **(C)**. Analyses were also performed for previously obtained histopathological samples (n = 4 for acute treatment, n = 5 for acute control) [27]. Scale bar 200 μm. Error bars indicate STDEV, and significance was calculated using one-way ANOVA (ns: p ≥ 0.05, **p < 0.01).

The blue color density of histopathological MT samples was measured to evaluate collagen deposition at wound sites (Figure 5). Based on the contrast analyses of chronic wounds on day 14 (Figures 5A,B) and acute wounds on day 9, collagen deposition was significantly lower (12.2 ± 0.09% and 23.7 ± 0.1%) in chronic wounds compared with treated acute wounds (43.2% ± 18.6%; p = 0.003 between treatment groups, p = 0.004 chronic control vs. acute treatment) (Figure 5C). Higher blood glucose levels (>23.4 mmol/L) correlated with lower collagen deposition (p < 0.01).

Histological evaluation revealed plump horizontally orienting fibroblasts and palely staining collagen in the regenerating dermal connective tissue in both control and treated samples, suggesting maturing fibrous tissue with subjectively scarce collagen formation as well as sparse to moderate neovascularization (Supplementary Material 2). One control wound exhibited granulation tissue and one treated wound immature collagenous connective tissue.

## Discussion

**FIGURE 6:**
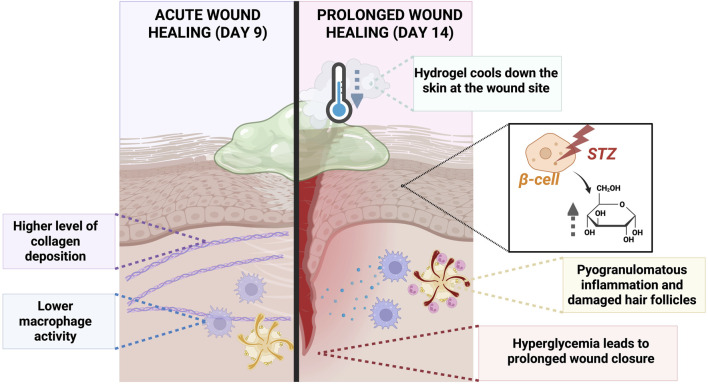
Graphical summary of the comparison between the acute wound model and prolonged wound healing model implemented in SKH1 mice.

In this study, diabetes was successfully induced in all tested SKH1 mice. Furthermore, successfully prolonged full-thickness wound healing was observed in all surgical animals when compared to our previously described acute full-thickness wound model with the same surgical procedure and mouse strain [27]. SKH1 mouse is an outbred, euthymic, and immunocompetent strain and, due to the hairless skin, suitable as a wound model [23]. Male mice were used for their susceptibility to STZ induced cytotoxicity in pancreatic islet β-cells compared to females [36]. This likely enabled the successful induction of diabetes in all mice (blood glucose concentration ≥15 mmol/L). Normal blood glucose concentration in C57BL/6J mice is between 5.5 and 11 mmol/L, which was used as a reference value [37]. Using a small needle and aspiration technique, no animals showed clinical symptoms due to failed STZ injection (The total number of STZ injections was 50, 5 per mouse). The repeated low STZ dose approach was chosen based on reported outcomes of single high-dose STZ (150–200 mg/kg IP), which induces a high acute mortality risk, often due to severe hypoglycemia and systemic toxicity [28]. The low-dose STZ approach only partially damages pancreatic islets, triggering an inflammatory process that causes the further loss of β-cell activity that ultimately results in insulin deficiency and hyperglycemia. The graphical summary comparing the outcomes of the previous acute wound model and prolonged wound healing model is presented in Figure 6.

In addition, animal welfare was closely monitored throughout the study by daily assessment of weight, behavior, and the risk of insulin dose-related hypoglycemia, all while maintaining on *ad libitum* diet. Since STZ induction has been reported to be equally diabetogenic to fed or fasted mice, fasting was not necessary in this model [38, 39]. This approach adheres to humane endpoints and ensures consistency across experimental conditions, facilitating reliable comparisons between prolonged and acute wound models under future standardized settings. However, it should be addressed that STZ induction produces hyperglycemic conditions similar to type 1 diabetes, while diabetic chronic wounds are often associated with type 2 diabetes due to metabolic syndrome, obesity, and insulin resistance. STZ induction of diabetes was selected in this case as it offers a controlled and non-transgenic approach, which helps to improve standardization when compared with genetically modified obese mouse strains or high-fat diet-based models alone. In future, a combined STZ induction and high-fat diet model could produce a diabetic chronic wound model with the characteristics of type-2 diabetes [40, 41].

In addition to blood glucose level monitoring, hematological parameters were measured at the end of the experiment. Based on the hematological parameters, a slight increase in the number of white blood cells, red blood cells and platelets were observed. However, these changes were not significantly different from our previous data [27] or reference values. A similar increase in these hematological values was observed in a study by Anggraeni et al. [42] where hematological parameters were studied in STZ induced diabetic mice with and without cogon grass treatment. However, most published *in vivo* models report inconsistent hematological data when comparing diabetic and healthy mice, making it difficult to draw definitive conclusions based on these parameters.

Higher blood glucose levels in SKH1 mice might have led to prolonged wound closure compared with similar size wounds in the acute wound healing model [27]. In prolonged wound healing models, wounds were only closed on day 14; this is 5 days longer than in acute wounds, which closed on day 9. The full-thickness wound creation was carried out as in our previous acute wound model reaching the muscle layer, partial destruction of which may lead to slower wound contraction. Similar observations have been made by Wyles et al. in which full-thickness wounds were created for C57BL/6 mice [43]. Chronic wounds were modeled by inducing oxidative stress with 3-amino-1,2,4-trizole and thiomalic acid as intraperitoneal injections, leading to slower wound contraction compared with non-injected mice. Although impaired wound closure is one characteristic in prolonged wound healing, wound closure in mice is affected by the *panniculus carnosus* and movement of the loose skin during surgical operation and wound covering. For this reason, other impaired wound healing-related factors, like re-epithelialization, reformation of extracellular matrix, angiogenesis and immunological changes are more reliable factors to study. The length of re-epithelialization and the thickness of the regenerating wound area were measured, which were not different to the acute wound healing model with or without model treatment. However, it is noteworthy that in the prolonged wound healing model, length and thickness values were measured on day 14 but on day 9 in the acute wound, correlating with the slower wound closure and healing. Reduced collagen deposition in quantitative analysis using MT-stained slides, along with histologically detected maturing fibrous tissue that exhibited subjectively scarce collagen formation on day 14, further attest to delayed healing, which might be related to the higher blood glucose levels that correlated with lower collagen deposition. Additionally, while the treatment model enhanced epithelialization compared to control in acute wounds, there was no difference in the prolonged wound healing setting. This underscores the importance of incorporating both acute and prolonged wound healing models in early preclinical studies to accurately assess the therapeutic potential of candidate treatments across diverse wound types.

Thermidas thermal imaging system was used to measure temperature changes in differently treated wounds and healthy skin. In general, temperature monitoring can non-invasively detect early diagnosis of prolonged inflammation, the state of angiogenesis and the total progress of wound healing. In addition, thermal analyses indicate treatment candidate effects on wound site which may reflect on healing outcome [44–46]. In this study, temperature was significantly decreased in the treated wounds compared with healthy skin for 6 days after wound creation. Extreme temperature changes may perturb the progression of wound healing for instance by affecting cell functionality and degradation of proteins [47–49]. However, these effects were not observed in the wound healing outcomes of this study. Interestingly, the high water content of the hydrogel-based model treatment lowered local wound temperature, suggesting potential as a first-aid cooling agent for wounds, such as burns or acutely inflamed wounds [50]. The thermal imaging system was successfully used to measure temperature in this *in vivo* model with sufficient sensitivity to detect temperature variations between wounded and healthy skin. To our knowledge, Thermidas has not previously been used in *in vivo* models, for which reason this study highlights potential novel applications for the thermal imaging instruments in research and clinical settings.

A key issue in diabetic prolonged or chronic wounds is disturbed blood vessel formation due to the effects of high blood glucose concentration on the endothelial cell functions [51]. This leads to insufficient oxygen and nutrient supply at the wound site and prolongs wound healing. According to Shaterian et al. [52], the progression of angiogenesis in a full-thickness acute wound model in C57/B16 mice treated with a collagen-based wound dressing showed the greatest presence of blood vessels between days 10 and 14 after which wound maturation begins. In our study, angiogenesis was evaluated based on the CD31-positive area in tissue sections. No significant differences were observed between prolonged and acute wound models in any treatment condition, and this similarity was also observed in the histological analysis. However, since the CD31 staining area was measured only after sacrifice of the animals (day 9 and 14 in the acute and prolonged wound healing models, respectively), a delay or a decrease in angiogenesis activity may only be inferred. Future studies should include all conditions in a single study and histologically compare angiogenesis on the same experimental day.

Another major factor in the progression of wound healing and activation of angiogenesis is the change in presence of pro-inflammatory macrophages into tissue regenerative ones, roughly categorized as M1 and M2 phenotypes [14, 53]. In diabetic wounds, due to the infection, and increased blood glucose concentration, the ratio between these phenotypes is disturbed, leading to persistence of M1 and prolonged inflammation [54]. Although the presence of specific macrophage phenotypes was not analyzed in this study, we observed significant increases in the macrophage-stained area (Iba-1) [55] with higher expression of the LZM enzyme, which may be related to enhanced macrophage activity [56], when compared to our previous acute wound model [27]. Increased LZM immunostaining may also be associated with increased presence of neutrophils at the wound site and/or in the pyogranulomatous inflammatory foci, which could lead to prolonged active neutrophilic inflammation [56]. Interestingly, in addition to a generally modest macrophage reaction to the NFC hydrogel, the prolonged wound healing model exhibited excessive pyogranulomatous inflammation affecting hair follicles. Granuloma formation was markedly more severe than that observed in our previous acute wound model [27] or the hair follicle granuloma formation typical of the SKH1 hairless mice [23]. This prolonged pyogranulomatous inflammation likely originated from the wounding trauma and corroborates the increased LZM immunostaining. These observations suggest that the diabetic full-thickness wound model more closely resembles the characteristics of prolonged, compromised wound healing than the previously published acute wound healing model.

The imbalance in macrophage activity in wound healing may lead to increased levels of reactive oxygen species damaging the ECM during re-modeling [57]. In addition, a hyperglycemic environment increases protease levels [53] and disturbs cell-level crosstalk between macrophages and fibroblasts that has a role in producing collagen at the wound site [58]. In this study, collagen deposition was significantly lower despite the treatment conditions when compared with the treated acute wounds indicating prolonged wound healing in diabetic mice. Furthermore, the markedly different outcomes observed between treatment groups suggest that prolonged wounds or wounds with chronic characteristics may require therapeutic strategies focused on enhancing cellular functionality, rather than solely providing mechanical support and an optimal wound bed environment. This study highlights the importance of creating comparable preclinical models in highly heterogenous wound research, which could reveal different physiological outcomes with varying treatment needs.

## Conclusion

To our knowledge, no previous published work has compared acute wound healing and diabetic prolonged wound healing in hairless SKH1 mice. This pilot study may show the suitability of SKH1 mice for studying full-thickness wound healing under different conditions. Diabetes was induced in all mice using repeated STZ induction without complications. The markedly prolonged wound closure time, elevated macrophage activity, and reduced collagen deposition reflected key features of chronic wounds. However, establishing a fully representative chronic wound model requires additional considerations, like reduced oxygen availability, and the potential influence of infection; these factors were not examined in this study. With careful monitoring, SKH1 mice may be used in future wound healing studies as acute and prolonged wound healing models that are highly tractable for STZ-induced diabetes. The sample size of this proof-of-concept study was deliberately small and relies on wound healing in SKH1 mice in previously published data. Therefore, studies with a larger sample size, including healthy control groups and other treatment candidates, should be performed in future. By offering the opportunity to generate standardized and reproducible *in vivo* models, the development of wound treatment candidates can be enhanced while adhering to the 3Rs principles.

## Data Availability

The raw data supporting the conclusions of this article will be made available by the authors, without undue reservation.
