# Supplementary material for: Towards a standardized diabetic prolonged wound healing model in hairless SKH1 mice
Source: Exp Biol Med (Maywood). 2026 Mar 20;251:10857. doi: 10.3389/ebm.2026.10857 (PMC13046712; doi:10.3389/ebm.2026.10857)
Supplement: Supplementary file 1 [file DataSheet1.docx]

Towards Standardized Diabetic Prolonged Wound Healing Model in Hairless SKH1 Mice

*Elle Koivunotko^1^, Julia Monola^1^, Chris S. Pridgeon^1^, Jere Linden^2,3^, Riina Harjumäki^1^, Emrah Yatkin^4^, Mari Madetoja^5^, Marjo Yliperttula^1^*

*^1^Division of Pharmaceutical Biosciences, Drug Research Program, Faculty of Pharmacy, University of Helsinki, 00790 Helsinki, Finland; ^2^Finnish Centre for Laboratory Animal Pathology, HiLIFE, University of Helsinki, Helsinki, Finland; ^3^Department of Veterinary Biosciences, Faculty of Veterinary Medicine, University of Helsinki, Helsinki, Finland; ^4^Central Animal Laboratory, University of Turku, Turku, Finland; ^5^Made Consulting Ltd, Tykistökatu 4b, 20520 Turku, Finland*

**RESULTS**

*Thermal image analyses showed clear temperature scaling between healthy skin and wound area until wound closure*

Thermal images were accompanied by a temperature scales: black-purple-blue colors indicated cooler temperatures (~24–33ºC), and green-yellow-red represented warmer temperatures (~34–40 ºC). The region-of-interest were chosen visually by comparing the color changes in the mouse and ensuring the wound location from the original images. The visual outcome of the thermal image is presented in Figure S1.


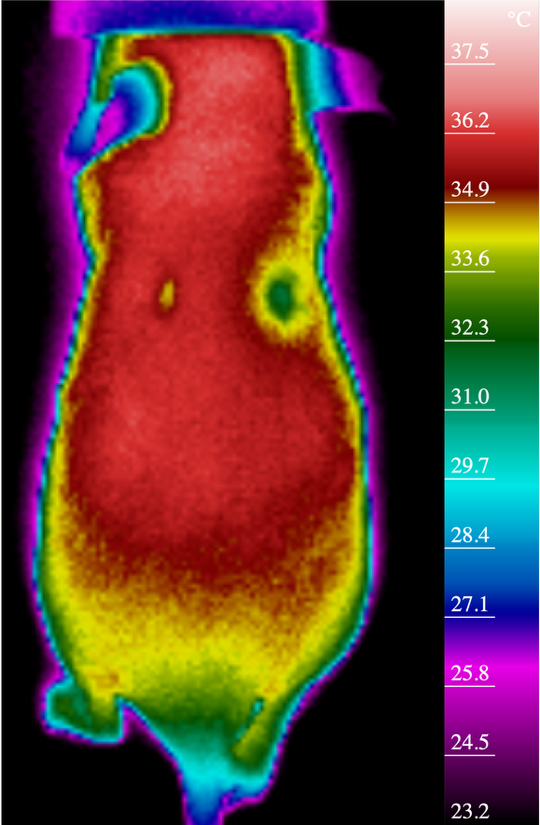


Figure S1 Example of the obtained thermal image with temperature scaling range. Image is taken on Day 2 after wound creation. The colors indicate the temperatures shown on the right side.

*Damaged hair follicles observed in histological HE- stained sections*

The Hematoxylin and Eosin (HE) stained sections of the prolonged full-thickness wound healing model with hairless SKH1 mice showed markedly severe presence of pronounced, focally extensive hypodermal and deep dermal pyogranulomatous inflammation affecting damaged hair follicles and sebaceous glands compared with the previously performed acute wound model with the same strain (1). This was most prevalent in the wound borders (Figure S2).


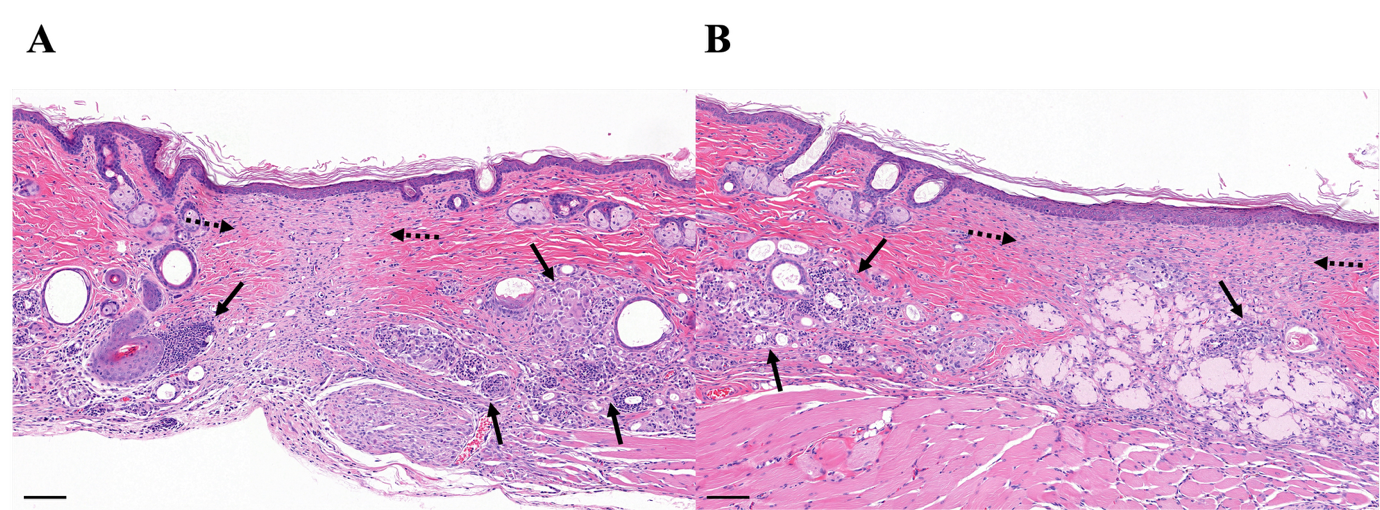


Figure S2. HE staining of focally extensive hypodermal and deep dermal pyogranulomatous inflammation (arrows) affecting damaged hair follicles in a control wound (A) and in a treated wound (B). Margins of regenerating dermal connective tissue in superficial dermis with plump horizontally orienting fibroblasts and palely staining collagen (dotted arrows). Scale bar 100 µm.

**REFERENCES**

1. Koivunotko E, Koivuniemi R, Monola J, Harjumäki R, Pridgeon CS, Madetoja M, et al. Cellulase-assisted platelet-rich plasma release from nanofibrillated cellulose hydrogel enhances wound healing. Journal of Controlled Release. 2024;368.
